# Thermometers

**Published:** 1861-01

**Authors:** 


**THERMOMETERS.**—Most persons know that sudden changes of weather endanger the health, and not unfrequently occasion the loss of valuable lives. These sudden and great changes often take place during the night. But it requires a day or two for the cold to get into the house, with the result that a person gets up, dresses, and gets into the street, before he discovers that it is fifteen, twenty, or thirty degrees (or even more) colder than it was the day previous; but before he has arrived at the knowledge of this fact, he has been chilled, or has started on a journey, or has got so far from home that a change of clothing is very inconvenient, if not almost impracticable. Franklin, Abbott Lawrence, Rachel the tragedienne, and other eminent personages, lost their lives by dressing for a temperature, or exposing themselves to a degree of coldness in the atmosphere, of which they were not conscious.

It is said of the Duke of Wellington, that he had every variety of garment; that his servant dare not bring any one of them to him, until he had gone himself to the window, hoisted it, and by the protrusion of his immense long nose out into the atmosphere, had calculated with this new feeler, the state of the weather, and the coat adapted to it. That he had such a nose we can testify, having seen it *on the outside of Windsor Castle!* on the occasion of Louis Philippe's visit there to her present Majesty. As to the variety of clothing, we speak from hearing. At all events, the Duke lived a long time, and the habit referred to was a wise one, by whomsoever practiced. The lesson is this: Judicious attention to the state of the thermometer, at least every morning, would answer a wise purpose, by acquainting us with the state of the weather out of doors, thus enabling us to dress in reference to it. Every family who can possibly afford it, should have a thermometer, of easy access in the lower hall, in each sitting-room, and a large one, with a red column, at the most northern exposure practicable, outside, in a situation easily accessible to every member of the family, without raising the window or opening the door. This would enable the servants to know of a morning how much of a fire to build. Fowler & Wells keep a good supply of these articles at 308 Broadway, New-York, at all prices, from half a dollar upwards, and will forward them safely to any address if the money accompanies the order. Those to be placed out of doors should be wholly of metal and glass. The round ones, dial-plate, with pointers, having a Fahrenheit and Reaumur's scale, are preferable on some accounts. Patented by V. Beaumont, 175 Center street, New-York. This notice is volunteered wholly for the convenience of our subscribers, good fellowship towards the parties named being thrown in ; for we must sometimes help one another without fee or reward. He who can't do that is a perfect nobody. A million such would not make the ninth part of a tailor, and, "they say," it takes nine of them to make a man.

